# Mulberry extract upregulates cholesterol efflux and inhibits p38 MAPK‐NLRP3‐mediated inflammation in foam cells

**DOI:** 10.1002/fsn3.3296

**Published:** 2023-03-08

**Authors:** Yuting Liu, Kefan Wang, Shuofei Yang, Guanhua Xue, Liming Lu

**Affiliations:** ^1^ Department of Vascular Surgery, Renji Hospital Shanghai Jiao Tong University School of Medicine Shanghai China; ^2^ Department of Immunology and Microbiology, Shanghai Institute of Immunology Shanghai Jiao Tong University School of Medicine Shanghai China

**Keywords:** foam cells, MAPK signaling pathway, mulberry, NLRP3 inflammasome, restenosis

## Abstract

The accumulation of foam cells in arterial intima and the accompanied chronic inflammation are considered major causes of neoatherosclerosis and restenosis. However, both the underlying mechanism and effective treatment for the disease are yet to be uncovered. In this study, we combined transcriptome profiling of restenosis artery tissue and bioinformatic analysis to reveal that NLRP3 inflammasome is markedly upregulated in restenosis and that several restenosis‐related DEGs are also targets of mulberry extract, a natural dietary supplement used in traditional Chinese medicine. We demonstrated that mulberry extract suppresses the formation of ox‐LDL‐induced foam cells, possibly by upregulating the cholesterol efflux genes ABCA1 and ABCG1 to inhibit intracellular lipid accumulation. In addition, mulberry extract dampens NLRP3 inflammasome activation by stressing the MAPK signaling pathway. These findings unveil the therapeutic value of mulberry extract in neoatherosclerosis and restenosis treatment by regulating lipid metabolism and inflammatory response of foam cells.

## Graphical abstract

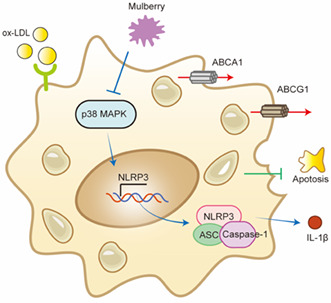

## 1 INTRODUCTION

Endovascular therapy has broad applications in clinical diagnosis and treatment of cardiovascular diseases (Sun, 2014). However, restenosis remains a major cause of target lesion failure after interventional therapy because 15.3% of patients with femoral popliteal artery disease develop in‐stent restenosis after stent implantation (Qato et al., 2018) despite the rapid development of drug treatment and surgical technology.

Histology, angiography, and intravascular imaging data all suggest that neoatherosclerosis is an important contributing factor for restenosis after vascular stent treatment (Park et al., 2012). Neoatherosclerosis is triggered by the activation of endothelial cells, followed by the recruitment of circulating monocytes which subsequently differentiate to monocyte‐derived macrophages and then macrophages foam cells. Foam cells are characterized by high intracellular lipid content and usually reside in the intima of artery hyperplasia (Funk et al., 1993). The late stage of neoatherosclerosis is accompanied by thin‐walled fibrous atherosclerosis and lipid‐rich neointima (Jinnouchi et al., 2017). Activated neointima cells secrete enzymes and chemicals that modify LDL, which in turn activate neointima cells and trigger various inflammatory signals (Pentikäinen et al., 2000). Thus, neoatherosclerosis plaques are featured by inflammation and lipid metabolic disorder.

NOD [nucleotide oligomerization domain]‐, LRR [leucine‐rich repeat]‐, and PYD [pyrin domain]‐containing protein 3 (NLRP3) containing inflammasome is an important part of innate immunity and plays essential roles in inflammation (Rathinam & Fitzgerald, 2016). NLRP3 is a key component of this multi‐protein complex because it interacts closely with apoptosis‐related dot‐like protein (ASC) and recruits the precursor of cysteinyl aspartate‐specific proteinase (caspase‐1; Zhen & Zhang, 2019). Previous studies revealed elevated expression of the NLRP3 inflammasome in human atherosclerotic arteries (Shi et al., 2021). In addition, a study from the Canakinumab Anti‐Inflammatory Thrombosis and Outcomes Study (CANTOS) showed that inhibition of IL‐1β induced by NLRP3 inflammation reduces the incidence of atherothrombotic events and inflammation in patients after myocardial infarction (Ridker et al., 2017), suggesting that NLRP3 inflammasome may be a promising therapeutic target for cardiovascular diseases including atherosclerosis and restenosis. However, the underlying mechanism remains unclear.

Foam cells play key roles in the development of neoatherosclerosis. They are macrophages with excessive influx of modified low‐density lipoprotein (LDL) and high accumulation of cholesteryl ester (Javadifar et al., 2021). This biological process can be reversed by cholesterol efflux which utilizes ATP binding cassette transporter A1 (ABCA1), ATP binding cassette transporter G1 (ABCG1), and scavenger receptor B1 (SR‐B1) to transport excess intracellular lipids to liver for degradation (Wang & Westerterp, 2020). A traditional Chinese medicine Yin‐xing‐tong‐mai decoction (YXTMD) has been used to treat atherosclerosis by activating the PPARγ‐LXRα‐ABCA1/ABCG1 pathway to enhance cholesterol efflux (Zheng et al., 2021).

Mulberry extract, a nutrition‐rich natural dietary supplement, has long been used in Chinese medicine (Jan et al., 2021). It has been shown to reduce lipid oxidative stress, inflammation, and lipids accumulation in liver and help ameliorate lipid metabolism disorders in a nonalcoholic fatty liver rat model (Memete et al., 2022). In addition, the ethanol extract of black mulberry reduces foam cell formation and inhibits the development of atherosclerotic plaque through the ox‐LDL‐PPARγ‐CD36 feed‐forward cycle (Liu et al., 2020). The total phenols and flavonoids in the ethanol extract of mulberry and its derivative components are sufficient to elicit protective effects on oxidative stress and inflammation of macrophages stimulated by lipopolysaccharide (Yu et al., 2021). Mulberry extract also contains a high concentration of biologically active compounds such as anthocyanins and flavonols, which inhibit the expression of inflammatory mediators induced by lipopolysaccharide in RAW264.7 cells and reduce the secretion of pro‐inflammatory cytokines such as interleukin (IL)‐6 and tumor necrosis factor (TNF)‐α (Jung et al., 2019).

In this study, we revealed that mulberry extract not only inhibits the formation of foam cells by upregulating the cholesterol efflux to inhibit intracellular lipid accumulation but also dampens NLRP3 inflammasome activation by stressing the MAPK signaling pathway. Collectively, our results underscore the therapeutic value of mulberry extract in restenosis treatment.

## 2 EXPERIMENTAL SECTION

### 2.1 Reagents

DMEM medium, fetal bovine serum (FBS), 100 μ/mL penicillin, and 100 μ/mL streptomycin were purchased from GIBCO company of the United States. Oxidized low‐density lipoprotein (20605es05; China Yeasen Biology). Cell counting kit‐8 (CCK‐8), DOJINDO, Japan; Oil Red O powder (ab42024259; Absin Biology); total RNA extraction kit, reverse transcription kit, RT qPCR Kit (10606es60, 11141es60, 11202es080; Yeasen Biology). Anti‐nlrp3 antibody (ab214185; Abcam); anti‐rabbit LgG (CST, 7074s); anti‐caspase‐1 antibody (ab138483; Abcam), anti‐ASC antibody (67824; CST); anti‐il‐1 β Antibodies (31202; CST); anti‐Phospho‐p38 MAPK antibody (4511; CST); anti‐p38 MAPK antibody (8690; CST).

### 2.2 Mulberry extract

Mulberry fruit, *Morus nigra*, was harvested at ripe stage from the Plantation of National Mulberry Orchard (Zhenjiang, Jiangsu province, China). Fruits were dried by freeze dryer (EYELA FDU‐2100) and milled into powder for further use. Two grams of mulberry fruit powder were mixed with hexane (1:10 w/v) and shaken continuously in an ultrasonic bath at 55°C for 1 h to remove lipids and fatty acids. Under the same conditions, the pellets were sonicated with 80% ethanol (1:3 w/v) to prepare a polyphenol‐rich extract. The extract was then incubated overnight at 4°C and sonicated again for 25 min. Next, the extraction was filtered through Whatman No. 1 filter paper. The residue was re‐extracted in the same way, combining three filtrates (5 mL each). The ethanol from the three filtrates was combined and evaporated under vacuum at 40°C to obtain the dry extract. The extracts were placed in plastic bottles and then stored at −20°C until use. For quality control, we provided the table of the substances identified in the *Morus nigra* extract by liquid chromatography–mass spectrometry (LC–MS).

### 2.3 Tissue collection

Arterial samples were taken from patients with amputation due to atherosclerotic disease, while control arteries were taken from healthy individuals after accidental amputation. All samples are from Shanghai Renji hospital affiliated to Shanghai Jiao Tong University School of Medicine and processed in the Biobank of Renji Hospital. All procedures were approved by the Research Ethics Committee of Renji Hospital.

### 2.4 Cell cultures

RAW 264.7 cells were purchased from the American Type Culture Collection (ATCC). Cells were cultured in DMEM (Gibco) supplemented with 10% fetal bovine serum (FBS; Gibco) plus 1% streptomycin in a humidified incubator at 5% CO_2_ air and 37°C. When the cells grow to 80%–90% of the area, subculture, and reserve.

### 2.5 Quantitative reverse transcription PCR (RT‐qPCR)

The total RNA of cells was extracted according to the instructions of Trizol kit, and the RNA concentration and A260/A280 ratio were measured by nano drop2000. cDNA was obtained by reverse transcription kit; the amount of mRNA was measured by RT‐qPCR kit, and three replicates were set in each group. Reaction conditions: pre‐denaturation at 95°C for 10 min, denaturation at 95°C for 15 s, annealing/extension at 60 for 30 s, a total of 40 cycles. use ΔΔ*C*_T_ to calculate the gene expression level. The specific formula is as follows: Δ*C*_t_ = *C*_t_ value of target gene – *C*_t_ value of GAPDH Gene; ΔΔ*C*_t_ = experimental group Δ*C*_t_ control group Δ*C*_t_; relative gene expression in the experimental group = $2^{-\Delta \Delta C_{t}}$.

**Table:** 

Project	Forward primer sequence (5′–3′)	Reverse primer sequence (5′–3′)
β‐Actin	GAAATCGTGCGTGACATTAA	AAGGAAGGCTGGAAGAGTG
NLRP3	CCATCGGCAAGACCAAGA	ACAGGCTCAGAATGCTCATC
Caspase‐1	CGTCTTGCCCTCATTATCTG	TCACCTCTTTCACCATCTCC
ASC	TGGATGCTCTGTACGGGAAG	CCAGGCTGGTGTGAAACTGAA
IL‐1β	TACGAATCTCCGACCACCACTACAG	TGGAGGTGGAGAGCTTTCAGTTCATATG
ABCA1	GCTTGTTGGCCTCAGTTAAGG	GTAGCTCAGGCGTACAGAGAT
ABCG1	GTGGATGAGGTTGAGACAGACC	CCTCGGGTACAGAGTAGGAAAG
SRB1	CGAAGTGGTCAACCCAAACGA	CCATGCGACTTGTCAGGCT
ABCA1	GCTTGTTGGCCTCAGTTAAGG	GTAGCTCAGGCGTACAGAGAT
ABCG1	GTGGATGAGGTTGAGACAGACC	CCTCGGGTACAGAGTAGGAAAG

### 2.6 Western blotting

The human artery or cultured cells were lysed in a lysis buffer containing RIPA and 1 mM PMSF for 30 min on ice. After centrifugation at 12,000 ×g for 15 min at 4°C, the supernatants were collected as total proteins in artery or cells. The protein concentrations were also determined with a BCA assay kit. Aliquots (50 μg) of protein samples were separated on 10% SDS‐PAGE and electro‐transferred to polyvinylidene fluoride membranes. The PVDF membranes were incubated with primary antibodies overnight at 4°C after being blocked with 5% nonfat milk for 1 h. The membranes were then washed with TBST (five times, 3 min each) and incubated with secondary antibodies for 1–2 h at 37°C. The protein bands were detected with an enhanced chemiluminescence system on Tanon 5200s. Densitometric analysis was conducted by ImageJ. Actin proteins were detected as a control.

### 2.7 Immunofluorescence

Tissue sections embedded in paraffin were cut into 4 μm sections, deparaffinized in xylene, and rehydrated in graded alcohol solution. Slides were blocked with 5% (v/v) donkey serum for 30 min. After washing with 1× PBS, cells were stained with rabbit anti‐NLRP3 and rabbit anti‐CD68 overnight at 4°C. After three washes in PBS, the immunoreactive products were visualized by incubation with an appropriate secondary antibody (1:400) and DAPI (1:500, 216276; Roche) to visualize cell nuclei. Confocal microscopy was conducted using an Olympus CX31 confocal microscope.

### 2.8 Oil Red O (ORO) staining

Lipid content was histologically assessed using ORO staining. RAW264.7 cells were plated in 24‐well plates and incubated with 50 μg/mL ox‐LDL with or without Mulberry extract for 24 h. The cells were washed two times with PBS slightly, fixed with 4% paraformaldehyde for 30 min and then stained with filtered ORO solution (in a 6:4 ratio of 0.5% ORO in ddH_2_O) for 60 min. Wash the cells with distilled water for 1–2 times, 1–2 min each time and rinse with 60% isopropyl alcohol. The lipid droplets in the macrophages were observed under the microscope and collected images.

### 2.9 Cell counting kit‐8 analysis

The cell counting kit‐8 (CCK‐8) assay was applied to measure cell viability. RAW264.7 cells were cultured in a 96‐well plate at a cell density of 5000 cells/well for 24 h. They were added with different concentrations of ox‐LDL or different concentrations of mulberry extract. After incubating for 24 h, 10 μL/well CCK‐8 is dissolved in 90 μL/well culture medium, 100 μL/well CCK‐8 working solution was added. The OD value at the wavelength of 450 nm was measured using the microplate reader 1 h later.

### 2.10 Cellular cholesterol assay

Cells were grown in 6‐well plate to approximately 2 × 10^6^ cells. The cells were washed twice with PBS to remove medium serum and re‐suspended in 0.1 mL of lysate per 1 × 10^6^ cells and incubated in room temperature for 10 min. Lysates were then heated at 70°C for 10 min followed by centrifugation at 2000 g for 5 min at room temperature. Supernatant was used for enzymatic assay. Total cholesterol assay kit (E1015; Applygen Technologies Inc) and free cholesterol assay kit (E1016; Applygen Technologies Inc) were used in cellular cholesterol assay. Working solution was prepared by mixing reagent R1 with reagent R2 in a 4:1 ratio. Dilute 5 mM cholesterol standard with anhydrous ethanol to 2500, 1250, 625, 312.5, 156, 78, 39 μmol/L and cover promptly. 190 μL of working solution was added to the microplate. To each microplate, 10 μL of each blank control solution (anhydrous ethanol), standard, and testing sample were added, respectively. Plates were sealed with membrane and incubated at 37°C for 20 min followed by spectrometry assay. OD value of each microplate was measured at wavelength of 550 nm. The cholesterol content was corrected for the concentration of protein per mg, and the standard curve was plotted, and the sample concentration was calculated. Free cholesterol concentration was measured by free cholesterol assay kits. Cholesteryl ester content was determined by subtracting FC from total cholesterol. Each test was performed in triplicate

### 2.11 Cell apoptosis analysis

Cell apoptosis was measured by using Annexin V‐FITC/PI apoptosis detection kit (BD556547). After treatment for 24 h, cells were collected, washed with PBS, and resuspended in binding buffer. Then, cells were incubated with 5 μL Annexin V‐FITC and 5 μL PI for 15 min at RT in the dark. A flow cytometer was used to analyze cell apoptosis. The rate of apoptosis is expressed as the percentage of annexin V cells (Annexin V ^+^ PI^−^) that are individually stained.

### 2.12 Metabolomics analysis

Accurately weigh 25 mg of sample and add 0.6 mL 2‐chlorophenylalanine (4 ppm) methanol (−20°C), vortex for 30 s in 2 mL EP tube, then add 100 mg glass beads, put them into the tissue grinder, and grind for 90 s at 60 Hz. Room temperature ultrasound for 15 min, 4 centrifugations at 138000 *g* at 4°C for 10 min, take 300 μL supernatant and filter through 0.22 μm membrane, and add the filtrate into the detection bottle. Use the rest of the samples for LC–MS detection.

Chromatographic separation was used with an ACQUITY UPLC® HSS T3 (150 × 2.1 mm, 1.8 μm; Waters) column maintained at 40°C. The temperature of the autosampler was 8°C. Gradient elution of analytes was carried out with 0.1% formic acid in water (C) and 0.1% formic acid in acetonitrile (D) or 5 mM ammonium formate in water (A) and acetonitrile (B) at a flow rate of 0.25 mL/min. Injection of 2 μL of each sample was done after equilibration. An increasing linear gradient of solvent B (v/v) was used as follows: 0–1 min, 2% B/D; 1–9 min, 2%–50% B/D; 9–12 min, 50%–98% B/D; 12–13.5 min, 98% B/D; 13.5–14 min, 98%–2% B/D; 14–20 min, 2% D‐positive model (14–17 min, 2% B‐negative model).

The ESI‐MSn experiments were used with the spray voltage of 3.5 and −2.5 kV in positive and negative modes, respectively. Sheath gas and auxiliary gas were set at 30 and 10 arbitrary units, respectively. The capillary temperature was 325°C. The Orbitrap analyzer scanned over a mass range of *m*/*z* 81–1000 for full scan at a mass resolution of 70,000. Data‐dependent acquisition (DDA) MS/MS experiments were performed with HCD scan. The normalized collision energy was 30 eV. Dynamic exclusion was implemented to remove some unnecessary information in MS/MS spectra.

### 2.13 Statistical analysis

Used online bioinformatics tools provided by DAVID (https://david‐d.ncifcrf.gov/).

Experimental data were presented as means ± SEM. *n* expresses the number of independent experiments or samples. Statistical analysis was performed by the one‐way analysis of variance (ANOVA) with the significance level set at *p* < .05 (GraphPad Prism 8).

## 3 RESULTS

### 3.1 Increased expression of NLRP3 in vascular tissues in restenosis

**FIGURE 1:**
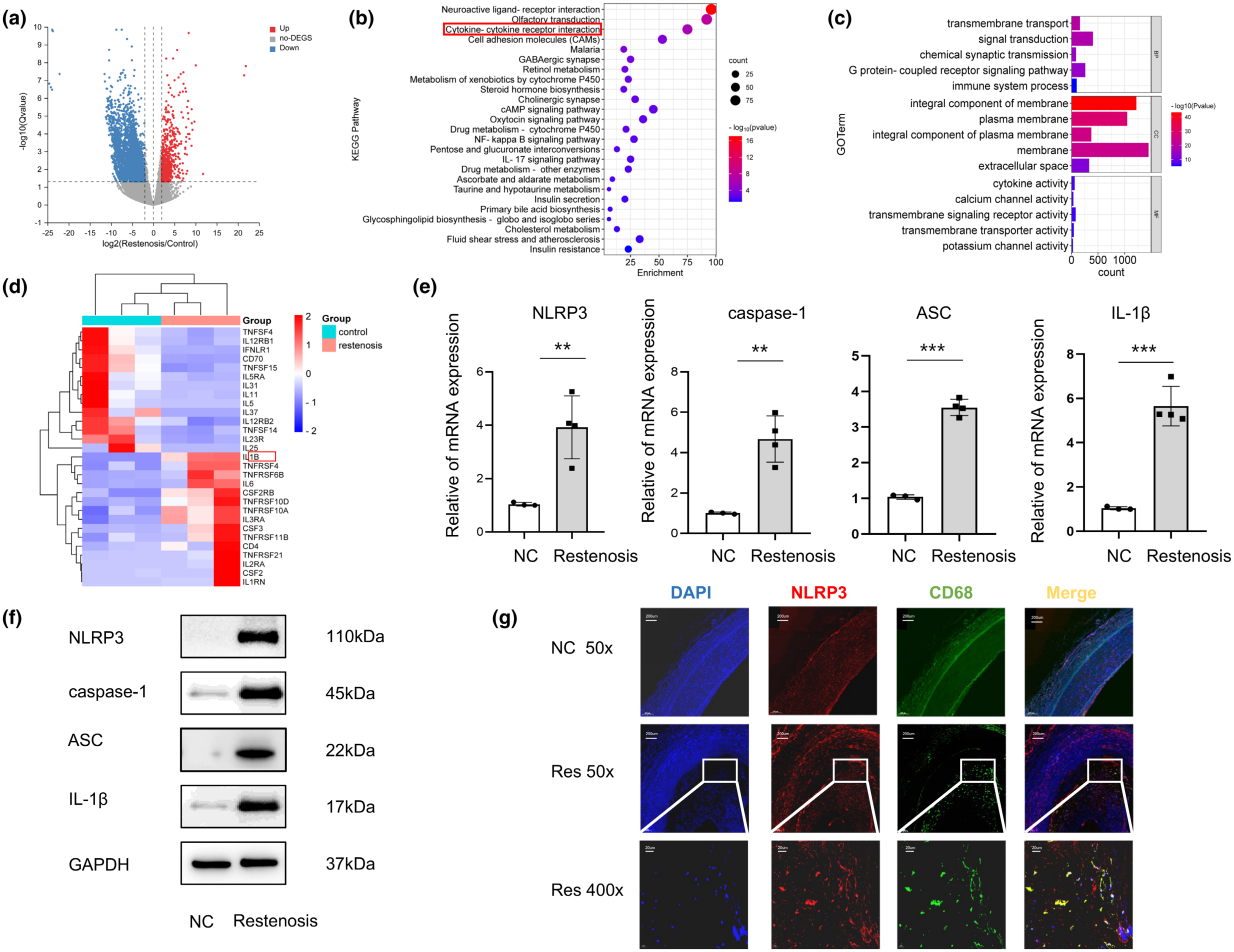
NLRP3 pathway is upregulated in neointima macrophages of restenosis patients. (a) Volcano plot (left) shows gene expression level in artery tissue of restenosis patients or healthy control with ‐log10 adjusted *p*‐value (*y*‐axis) versus log2 fold‐change (*x*‐axis). Up‐ and down‐regulated DEGs (with adjusted *p*‐value < .05 and log2 fold‐change > 2) are labeled with red and blue dots, respectively. Genes with no significant change are labeled with gray dots. (b) KEGG pathway enrichment analysis of the DEGs reveals the top enriched biological processes in artery tissue of restenosis patients compared to healthy control. (c) GO enrichment analysis of DEGs (BP, biological process; CC, cellular component; MF, molecular function). (d) Heat map shows differentially expressed genes (with adjusted *p*‐value < .05 and log2 fold‐change > 2) between restenosis patients or healthy control (*n* = 3). (e) The mRNA expression levels of NLRP3, caspase‐1, ASC and IL‐1β in artery tissue of restenosis patients or healthy control were validated by RT‐qPCR. Relative expressions were normalized to β‐Actin (*n* = 4 per group). (f) Protein expression of NLRP3, caspase‐1, ASC, and IL‐1β were determined by Western blotting. (g) Expression of NLRP3 and CD68 in normal and restenotic artery by immunofluorescence. Red, NLRP3; green, CD68; blue, DAPI nuclear staining. Compared with normal arterial tissue, **p < .01, ***p < .001. FC, Fold change; NC, Normal Control; Res, Restenosis.

To understand the unique transcriptional network in restenosis, we used RNA‐seq analysis to compare the transcriptome of artery tissue from restenosis patients and healthy controls undergoing lower‐limb amputation. The volcano map (Figure 1a) shows a total of 3429 differentially expressed genes (DEGs; logFC > 2 and *p* value < .05), among which 605 DEGs being upregulated and 2824 DEGs downregulated in restenosis patients compare to healthy control. Further Gene Ontology (GO) and Kyoto Encyclopedia of Genes and Genomes (KEGG) analyses on the DEGs (Figure 1b,c) reveal enriched pathways in cytokine–cytokine receptor interaction and biological processes immune system process (highlighted in red box). Heat map shows genes in cytokine–cytokine receptor interaction, such as interleukin‐1 (IL‐1), are upregulated (Figure 1d). IL‐1 is a common pro‐inflammatory cytokine that plays a key role in the innate immune response (Dinarello, 2011).

Since the NLRP3 inflammasome is an innate immune signaling complex and a key mediator of IL‐1 family cytokine production (Grebe et al., 2018), we tested its mRNA level in restenosis (*n* = 4) and normal artery (*n* = 3) tissues by RT‐qPCR. The results showed that NLRP3, caspase‐1, ASC, and IL‐1β mRNA were all significantly increased (Figure 1e). Moreover, the NLRP3 protein level also markedly increased upon restenosis treatment compared with normal control group (Figure 1f). Fluorescence in situ hybridization confirmed the elevated signal of NLRP3 in restenosis artery tissue accompanied by increase filtration of CD68^+^ macrophage. Importantly, the co‐localization of NLRP3 and CD68 suggests that NLRP3 mainly expresses in neointima macrophages (Figure 1g). These results indicate a correlation between NLRP3 expression, especially in macrophages, and restenosis.

### 3.2 Active compounds in mulberry extract target genes involving in lipid metabolism and foam cell formation

**FIGURE 2:**
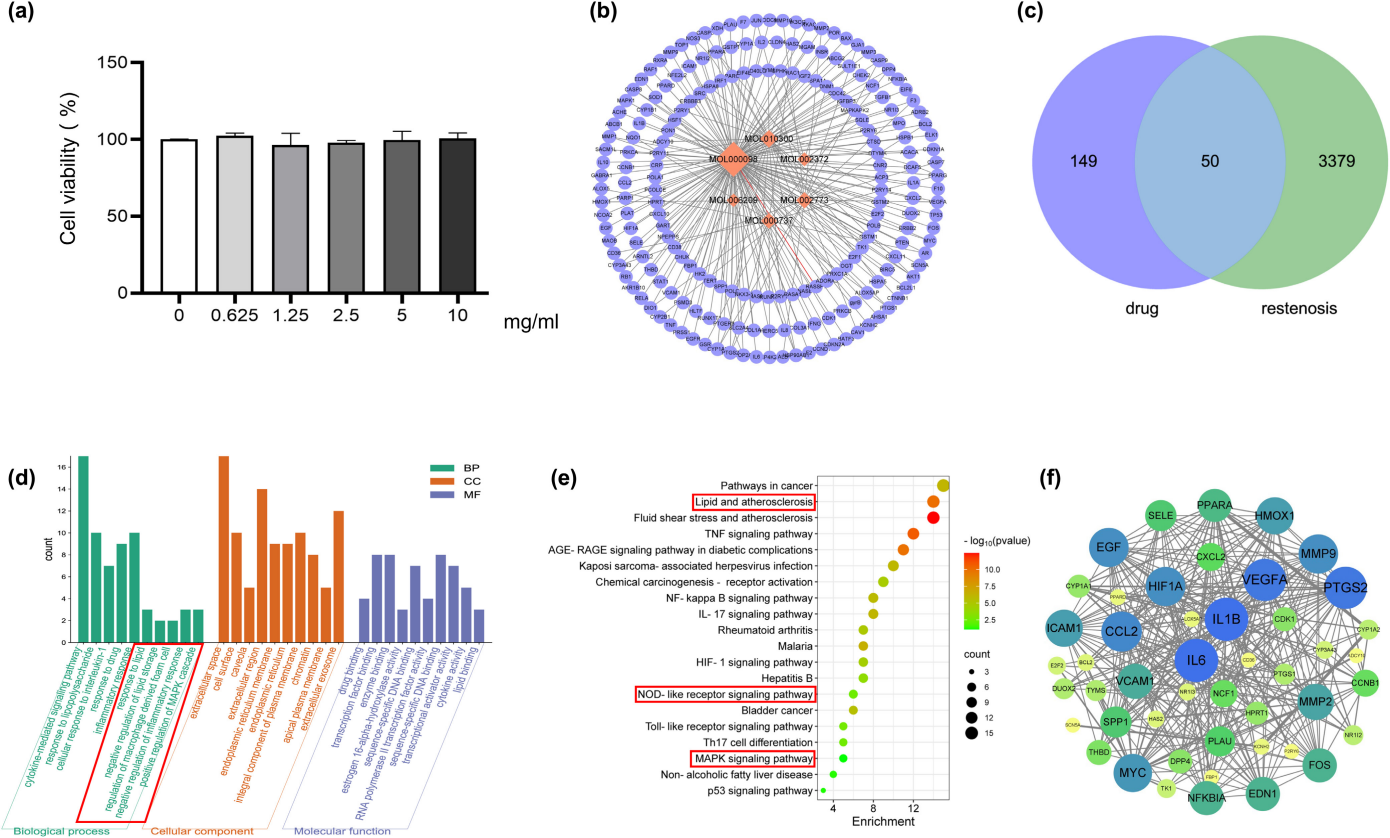
Active compounds of mulberry extract target anti‐restenosis genes. (a) RAW 264.7 macrophages were treated with mulberry extract (0, 0.625, 1.25, 2.5, 5, and 10 mg/mL) for 24 h, and cell viability was measured by CCK‐8 method. (b) Predicted network of targets of mulberry‐derived compounds using Traditional Chinese Medicine Systems Pharmacology Database. Orange nodes represent the active compounds, purple nodes the predicted targets, and edges indicate the interactions between compounds and targets. The size of nodes is proportional to the degree of interaction. (c) Venn diagram shows overlapping genes of predicted targets of mulberry compounds and genes downregulated in restenosis tissue. (d) KEGG pathway enrichment analysis and (e) GO enrichment analysis of the overlapping genes in (c). Pathways involve in lipid metabolism and inflammation are highlighted with red box. (f) Protein–protein interaction (PPI) network analysis of the overlapping genes by Cytoscape. Node size is proportional to the degree of interaction.

Mulberry leaf and fruit extracts have long been used as traditional Chinese medicines to improve liver health by regulating glucose and lipid metabolisms (Lim et al., 2013). To evaluate the benefit of mulberry fruit *Morus nigra* extract in restenosis, we first tested its cytotoxicity on RAW264.7 macrophage. RAW264.7 cells were treated with different concentrations (0, 0.625, 1.25, 2.5, 5, 10 mg/mL) of mulberry extract for 24 h and the proliferation were measured by Cell Counting Kit‐8 (CCK‐8). We found that mulberry extract had no toxic effect on RAW264.7 cells up to the concentration of 10 mg/mL, (Figure 2a). Therefore, all subsequent experiments used 10 mg/mL as the highest concentration. Based on data obtained from the Traditional Chinese Medicine Systems Pharmacology Database (TCMSP: http://tcmspw.com/tcmsp.php), we identified six active compounds of mulberry extract with the criteria of OB ≥30% and DL ≥0.18 (Table 1). In PubChem database, which provides information on the activities of chemical molecules in biological assays, we found 196 potential targets of these compounds. Next, we established a compound–target network using Cytoscape software to illustrate the interaction between these active compounds and their targets. Our network consists of 203 nodes which represent active compounds or their potential targets and 231 edges which indicate the interaction between the nodes (Figure 2b). These targets were compared with the 3429 DEGs, and 50 overlapping genes (Table 2) were chosen for further study to examine the mechanism and anti‐restenosis efficacy of mulberry extract (Figure 2c). We also applied KEGG pathway enrichment (Figure 2d) and GO analysis (Figure 2e) on these anti‐restenosis targets and found that they are mainly involved in biological processes related to lipid metabolism and atherosclerosis such as inflammatory response, negative regulation of lipid storage, and negative regulation of macrophage‐derived foam cell differentiation. We then used Cytoscape software to optimize and visualize the protein–protein interaction (PPI) network of these genes provided by the STRING database (https://string‐db.org/). PPI is defined by interaction scores (medium: >0.4) (Figure 2f). Intriguingly, IL‐1β is one of the key hubs in the cluster (Table 3). The findings show that mulberry extract is non‐toxic and could regulate inflammatory response and lipid metabolism in foam cells.

**TABLE 1:** Six active compounds of Mulberry.

MOL ID	Name	MW	OB (%)	HL	Structure
MOL010300	dIDP	446.74	41.08	1.96	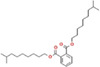
MOL002372	(E,E,E,E)‐Squalene	410.8	33.55	3.15	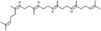
MOL006209	Cyanin	411.66	47.42	2.72	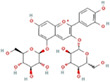
MOL000737	Morin	302.25	46.23	15.5	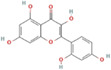
MOL002773	Beta‐carotene	536.96	37.18	4.36	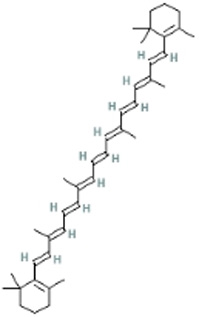
MOL000098	Quercetin	302.25	46.43	14.4	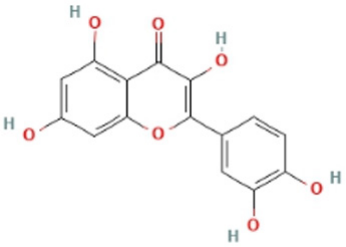

Abbreviations: DL, drug‐likeness; MW, molecular weight; OB, oral bioavailability.

**TABLE 2:** Fifty potential target genes of mulberry extract therapy for restenosis.

No.	Target	Symbol	Entrez ID
1	Prostaglandin G/H synthase 2	PTGS2	5743
2	Prostaglandin G/H synthase 1	PTGS1	5742
3	Dipeptidyl peptidase IV	DPP4	1803
4	Endothelin‐1	EDN1	1906
5	Platelet glycoprotein 4	CD36	948
6	Vascular endothelial growth factor A	VEGFA	7422
7	Apoptosis regulator Bcl‐2	BCL2	596
8	72 kDa type IV collagenase	MMP2	4313
9	Heme oxygenase 1	HMOX1	3162
10	Cytochrome P450 3A4	CYP3A43	64,816
11	Cytochrome P450 1A2	CYP1A2	1544
12	Myc proto‐oncogene protein	MYC	4609
13	Aldose reductase	AKR1B10	57,016
14	Potassium voltage‐gated channel subfamily H member 2	KCNH2	3757
15	Sodium channel protein type 5 subunit alpha	SCN5A	6331
16	Gamma‐aminobutyric acid receptor subunit alpha‐1	GABRA1	2554
17	Proto‐oncogene c‐Fos	FOS	2353
18	Urokinase‐type plasminogen activator	PLAU	5328
19	Matrix metalloproteinase‐9	MMP9	4318
20	Pro‐epidermal growth factor	EGF	1950
21	Interleukin‐6	IL6	3569
22	NF‐kappa‐B inhibitor alpha	NFKBIA	4792
23	Hypoxia‐inducible factor 1‐alpha	HIF1A	3091
24	Cell division control protein 2 homolog	CDK1	983
25	Cytochrome P450 1A1	CYP1A1	1543
26	Intercellular adhesion molecule 1	ICAM1	3383
27	Interleukin‐1 beta	IL1B	3553
28	C‐C motif chemokine 2	CCL2	6347
29	E‐selectin	SELE	6401
30	Vascular cell adhesion protein 1	VCAM1	7412
31	Dual oxidase 2	DUOX2	50,506
32	Nuclear receptor subfamily 1 group I member 2	NR1I2	8856
33	G2/mitotic‐specific cyclin‐B1	CCNB1	891
34	Thrombomodulin	THBD	7056
35	Arachidonate 5‐lipoxygenase	ALOX5AP	241
36	Neutrophil cytosol factor 1	NCF1	653,361
37	Hyaluronan synthase 2	HAS2	3037
38	C‐X‐C motif chemokine 2	CXCL2	2920
39	Nuclear receptor subfamily 1 group I member 3	NR1I3	9970
40	Peroxisome proliferator‐activated receptor alpha	PPARA	5465
41	Peroxisome proliferator‐activated receptor delta	PPARD	5467
42	Osteopontin	SPP1	6696
43	Transcription factor E2F2	E2F2	1870
44	Purinergic receptor P2Y1	P2RY6	5031
45	Hypoxanthine‐guanine phosphoribosyltransferase	HPRT1	3251
46	Thymidylate synthase	TYMS	7298
47	Adenylate cyclase type 10	ADCY10	55,811
48	Fructose‐1,6‐bisphosphatase	FBP1	2203
49	Thymidine kinase, cytosolic	TK1	7083
50	Cannabinoid receptor 2	CNR2	1269

**TABLE 3:** Top 10 high‐degree genes in the PPI network.

No.	Target	Symbol	Degree
1	Interleukin‐1 beta	IL1B	30
2	Interleukin‐6	IL6	29
3	Prostaglandin G/H synthase 2	PTGS2	28
4	Vascular endothelial growth factor A	VEGFA	28
5	C‐C motif chemokine 2	CCL2	26
6	Hypoxia‐inducible factor 1‐alpha	HIF1A	25
7	Epidermal growth factor receptor	EGF	25
8	Matrix metalloproteinase‐9	MMP9	25
9	Myc proto‐oncogene protein	MYC	24
10	Heme oxygenase 1	HMOX1	23

### 3.3 Mulberry extract inhibits the expression of NLRP3 via the MAPK signaling pathway in foam cells

**FIGURE 3:**
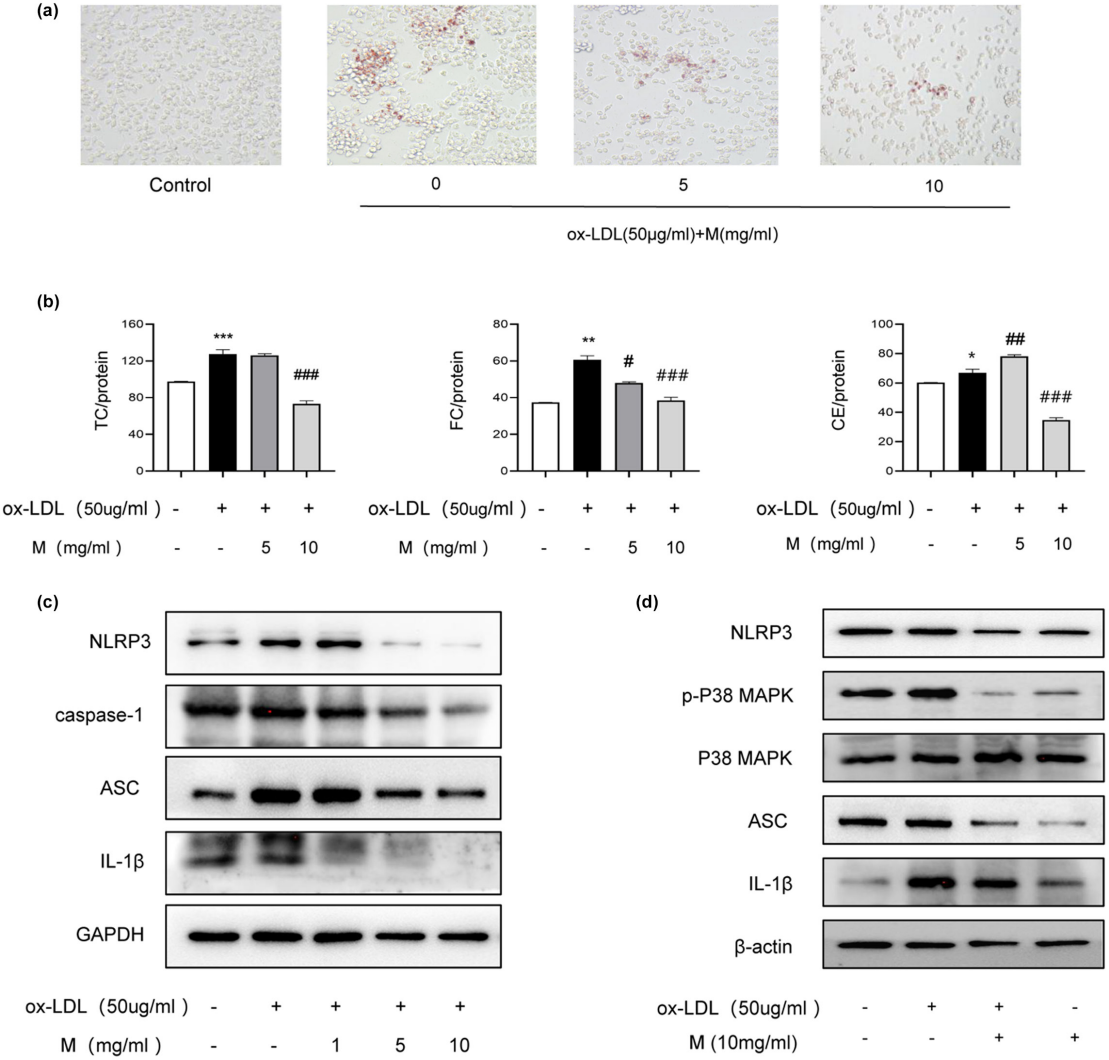
Mulberry extract inhibits the expression of NLRP3 in foam cells via MAPK signaling pathway. Foam cells were induced by treating RAW264.7 macrophages with 50 μg/mL ox‐LDL for 24 hours. The cells were then given mulberry extract at low (5 mg/mL) or high concentration (10 mg/mL) as indicated. Following foam cell features were monitored. (a). Intracellular lipid level measured by Oil Red O staining. (b). Cholesterol ester content. (TC, total cholesterol; FC, free cholesterol; CE, cholesterol ester) qualified by cellular cholesterol assay of foam cells with or without treatment of mulberry extract as indicated. (c). Induced foam cells were treated with different concentrations of mulberry extract (0, 1, 5, 10 mg/mL) for 24 h and protein levels of NLRP3, caspase‐1, ASC, and IL‐1 β were determined by Western blotting. (d) The protein levels of NLRP3, ASC, p‐p38, p38 MAPK, and IL‐1β protein levels of cells undergoing indicated treatments were determined by Western blotting. Graph bars represent qualification of flow cytometry results (Data shown are mean ± SEM. Statistical significance was determined by two‐way ANOVA with correction for multiple comparisons. *p < .01, **p < .001, ***p < .0001, ##*p* < .01 and ###*p* < .001.

The accumulation of lipids in macrophages and foam cell formation are closely associated with the development of atherosclerosis (Liu et al., 2021). In order to study the effect of mulberry extract on foam cell formation, we first established an in vitro inducible foam cell model (Zhang et al., 2020). Raw264.7 macrophages were induced with 50 μg/mL oxidized low‐density lipoprotein (ox‐LDL) for 24 hours to form foam cells. We then used Oil Red O staining to detect the lipid droplets in cytoplasm, a major feature of foam cells, and found accumulation of intracellular lipid in induced cells (Figure 3a, compare left two panels). Increased intracellular cholesterol ester content (cholesterol ester = total cholesterol‐free cholesterol) detected by the total cholesterol and free cholesterol enzyme quantification method further confirmed the successful formation of foam cells (Figure 3b). We then treated these ox‐LDL‐induced foam cells with either low (5 mg/mL) or high concentration (10 mg/mL) of mulberry extract for 24 h to examine the intracellular lipid content using Oil Red O staining. Intriguingly, our results show that ox‐LDL‐induced foam cells treated with both high and low concentration of mulberry extract contain significantly less lipid droplets compared with the no‐treatment cells (Figure 3a, compare right three panels). Consistently, the mulberry extract‐treated cells also show decreased levels of both total cholesterol and cholesterol ester (Figure 3b). We also observed that this inhibition is dose‐dependent (Figure 3b, compare lanes 3 and 4). These results indicate that mulberry extract has the ability to inhibit ox‐LDL‐induced RAW264.7 macrophage foam cell production by suppressing intracellular lipid accumulation.

Since the lipid content of foam cells is associated with inflammation, we aimed to explore whether mulberry extract inhibits the inflammasome activity in these cells. To this end, we utilized the same ox‐LDL‐induced foam cells. After induction, we divided the cells into control, 1, 5, and 10 mg/mL mulberry extract treatment groups and proteins involved in inflammasome activity were detected by Western blotting. Our results showed that upon induction, the protein levels of NLRP3, caspase‐1, ASC, and IL‐β all markedly increased. In contrast, foam cells treated with mulberry extract showed dosage‐dependent downregulation of NLRP3, caspase‐1, ASC, and IL‐1β (Figure 3c).

Given that the MAPK signaling pathway plays essential role in various cardiovascular diseases and has been shown to regulate NLRP3 (Zhao et al., 2019), we further explored the effects of mulberry extract on this pathway. GO analysis revealed that biological processes enriched in anti‐restenosis targets are also involved in positive regulation of MAPK cascade and KEGG pathway enrichment analysis confirmed that MAPK signaling pathway is the principal pathway on these targets (Figure 2d,e). To test the efficacy of mulberry extract on MAPK pathway, macrophages were pretreated with mulberry extract for 1 h, and after ox‐LDL induction, protein level of p38‐MAPK and MAPK were determined by Western blotting. The results demonstrate that cells treated with mulberry extract has reduced expression of p38‐MAPK (Figure 3d). Therefore, mulberry extract may regulate NLRP3 inflammasome through p38 MAPK signaling pathway.

### 3.4 Mulberry extract stimulates cholesterol efflux and inhibits apoptosis of foam cells

**FIGURE 4:**
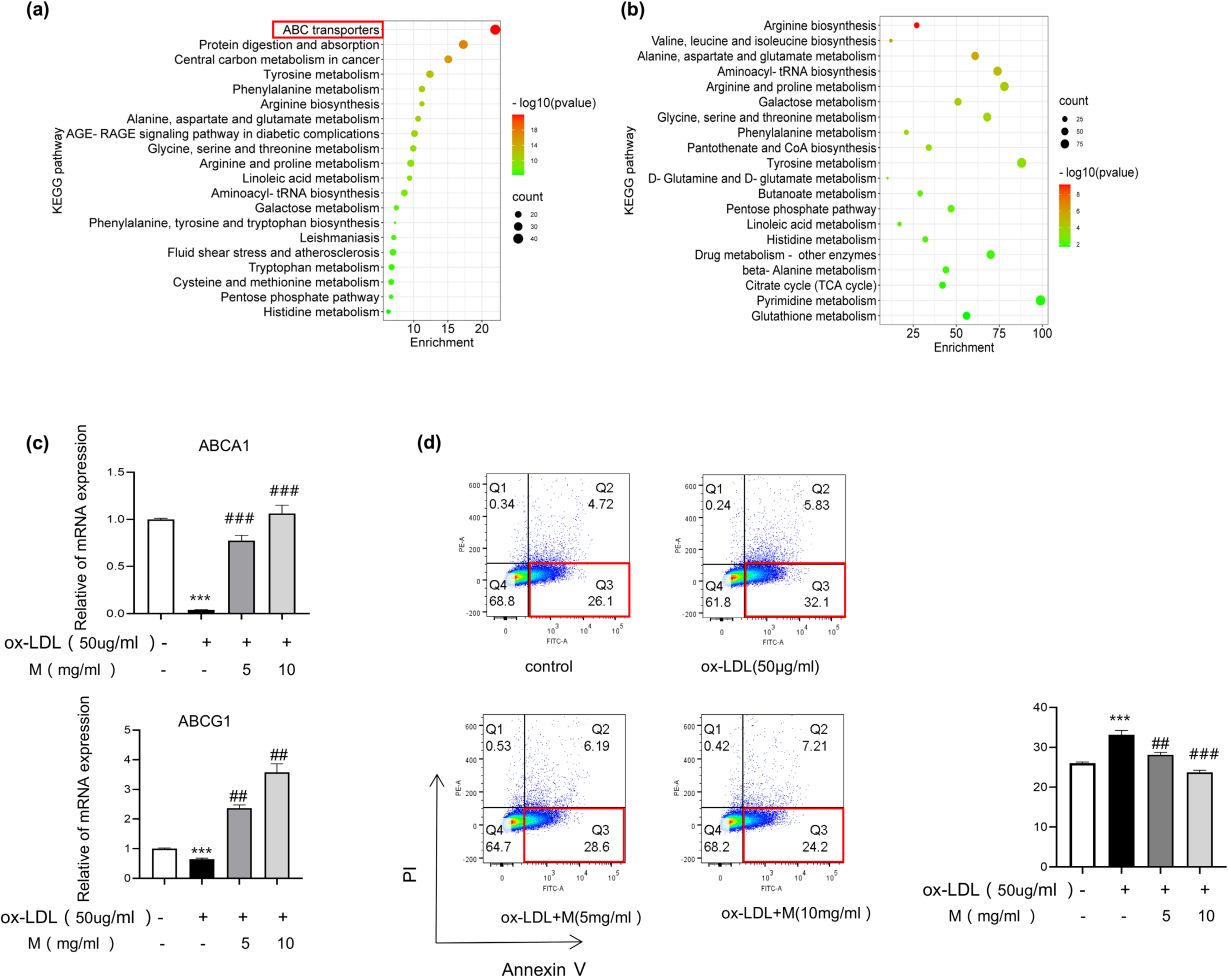
Mulberry extract treatment elevates cholesterol efflux and inhibits lipid accumulation and apoptosis in foam cells. (a) Pathways enrichment analysis of combined parameters of mulberry extract‐related metabolites and anti‐restenosis genes identified in this study using MetaboAnalyst. Cholesterol efflux pathway is highlighted by red box. (b) Metabolism pathways analysis using MetaboAnalyst. (c) The mRNA expression levels of ABCA1 and ABCG1 in induced foam cells with or without mulberry treatment as indicated were validated by RT‐qPCR. Relative expressions were normalized to β‐Actin gene. (d) Apoptosis of induced foam cells with or without mulberry treatment as indicated was detected using Annexin V flow cytometry (representative data are shown from three independent experiments). Statistical significance was determined by two‐way ANOVA with correction for multiple comparisons. ***p < .001, ****p < .0001, ##*p* < .01 and ###*p* < .001).

To further understand the mechanism of mulberry extract, we applied liquid chromatography–mass spectrometry (LC–MS) based untargeted metabolomics analysis and identified 727 related metabolites. These metabolites and the anti‐restenosis genes found in this study were then subjected to all pathways and metabolism pathways analysis by MetaboAnalyst (https://www.metaboanalyst.ca/; Figure 4a,b). The results show that the ABC transporters pathway is among the top 20 most enriched pathways. ABCA1 and ABCG1 are members of ABC transporters and mediate cholesterol efflux (Westerterp et al., 2018). Next, we used RT‐qPCR to detect the transcriptional expression of ABCA1 and ABCG1 in ox‐LDL‐induced foam cells with or without treatment of mulberry extract and found significant upregulation of both ABCA1 and ABCG1 (Figure 4c). Our result suggests that mulberry extract may stimulate cholesterol efflux mediated by ABCA1 and ABCG1 in foam cells to suppress their lipid accumulation. We also evaluated the effect of mulberry extract on foam cell apoptosis by Annexin V flow cytometry. Compared with that of control group, treatment with 50 μg/mL ox‐LDL for 24 h significantly increases the percentage of apoptotic cells. In contrast, we observed a dose‐dependent reduction of foam cell apoptosis with pretreatment of mulberry extract. The percentage of apoptotic cells drops form 45.1% in untreated cells to 34.9% in cells treated with 10 mg/mL mulberry extract (Figure 4d). The results altogether demonstrate the efficacy of mulberry extract in controlling cholesterol efflux and reducing apoptosis.

## 4 DISCUSSION

Foam cells, a group of lipid‐rich macrophages, play essential roles in the development of restenosis. In this study, we found that mulberry extract, a natural dietary supplement used in traditional Chinese medicine, inhibits the formation of foam cells by stimulating the cholesterol efflux, and provides a new therapeutic strategy for the treatment of restenosis. Our mechanistic studies by RNA‐sequencing and network pharmacological analysis of overlapping genes regulated by both mulberry extract and restenosis reveal that the reduction in lipid deposition and attenuation of inflammation in foam cells may be the future directions for preventing restenosis and its clinical manifestations.

The main mechanical feature of restenosis after endovascular treatment is insufficient stent expansion or fracture. Meanwhile, local inflammation leads to invasive neointimal hyperplasia and neoatherosclerosis (Shlofmitz et al., 2019). During the development of restenosis, macrophages infiltrate in neointima and eventually aggregate near the surface of the stent and cause neoatherosclerosis that is not related to the original atherosclerotic tissue (Otsuka et al., 2015). Subsequently, these fat‐laden macrophages, or foam cells, form atherosclerotic plaques, which can evolve into thin‐walled atherosclerotic plaques with the risk of plaque rupture and occasional calcification (Otsuka et al., 2015). The transformation of macrophages into foam cells is characterized by lipid droplet formation and massive lipid accumulation (Chistiakov et al., 2016). To reverse this biological process, cholesterol transporters such as ABCA1‐ and ABCG1‐mediated cholesterol efflux from macrophages to extracellular lipid acceptors. ABCA1 and ABCG1 deficiency increases foam cell formation and accelerates the development of atherosclerosis in mice. Several Chinese medicines are shown to promote cholesterol efflux by regulating the PPARγ‐LXRα‐ABCA1/ABCG1 pathway including Chrysin (Wang et al., 2015), Yin‐xing‐tong‐mai decoction (Zheng et al., 2021) and Qing‐Xue‐Xiao‐Zhi formula (Li et al., 2021). However, it is difficult to identify the active ingredient in these multi‐ingredient medicines and their anti‐inflammatory mechanism remains to be elucidated. Statins, as cholesterol‐lowering drugs, are effective in reducing cardiovascular disease and mortality in patients at high risk of cardiovascular disease. (Robson, 2008) Nevertheless, it may cause side effects including rhabdomyolysis and liver dysfunction (Ruscica et al., 2023). In contrast, mulberry, *Morus nigra*, is a nutrition‐rich dietary source and mulberry extract has no adverse health effect on human. Mulberry has been used in traditional Chinese medicine for centuries and increasing number of recent studies also show that mulberry fruit or leaf extracts are effective in boosting immunity, lowering blood glucose, and promoting metabolism (Zhou et al., 2017). Our results, for the first time, show that mulberry extract treatment increases the expression of cholesterol efflux genes ABCA1/ ABCG1 and inhibits lipid droplet formation and lipid deposition in foam cells. Interestingly, our metabolic pathway analysis of mulberry targets in restenosis‐related genes reveals a significant enrichment of arginine biosynthesis. L‐arginine, as a substrate for intracellular NO synthesis, has a variety of biological functions such as improving endothelial dysfunction and treating atherosclerosis (Li et al., 2019). A study in diabetes shows that oral L‐arginine promotes NO synthesis and enhances vascular function (Kohli et al., 2004). In the future, we will further explore the role of mulberry extract in improving the function of endothelial cells as a new avenue to treat restenosis.

There are studies indicate a link between lipid and inflammation (Li et al., 2005). The two factors may establish a positive feedback mechanism and synergistically contribute to disease progression. For example, lipid accumulation in Abca1/g1‐deficient myeloid cells promotes the activation of NLRP3 inflammasome (Westerterp et al., 2018). Abnormal lipid metabolism also causes increased expression of pro‐inflammatory cytokines and activating inflammation in atherosclerotic plaques (Westerterp et al., 2013). Our study used bioinformatic analyses of restenosis‐related genes and predicted mulberry extract targets to identify potential anti‐restenosis genes regulated by mulberry extract. The most noteworthy of these targets include genes associated with inflammation such as IL‐1β, IL‐6, CD36, HIF‐1α, MMP2, ICAM‐1, VCAM‐1, and VEGF. CD36 participates in phagocytosis of apoptotic cells, pathogen recognition, and regulation of low‐density lipoproteins. It also contributes to inflammatory responses and thrombotic diseases (Silverstein & Febbraio, 2009). MMP‐2 is a multifunctional protein which expression is elevated in many cardiovascular pathologies (e.g., myocardial infarction, hypertensive heart disease) where tissue remodeling and inflammatory responses are perturbed (Hardy et al., 2018). Among the targets are also IL‐1β which plays important roles in angiogenesis by synergistically inducing the production of VEGF with TNF and IL6. The expression of IL‐1β is dependent on the activation of NLRP3 because NLRP3 inflammasome cleaves pro‐caspase‐1 into the active form caspase‐1, thereby promoting IL‐1β maturation and secretion (Agostini et al., 2004). IL‐1β in turn regulates the initiation and amplification of inflammatory process. Intriguingly, our RNA‐seq and florescent imaging data reveal not only the increased levels of IL‐1β and NLRP3 in restenosis artery tissue but also a co‐localization of NLRP3 with CD68^+^ macrophages, suggesting the regulatory roles inflammasomes in foam cells play in the development of restenosis. We also found that mulberry extract is effective in suppressing the activation of NLRP3 inflammasome. The mRNA and protein expression of NLRP3 in mulberry extract‐treated foam cells are significantly decreased in a dosage‐dependent manner, making mulberry extract a promising therapeutic treatment for restenosis patients.

Protein kinases, including JNK, p38 MAPK, and ERK, are activated in response to various stresses (Kim & Choi, 2015). P38 MAPK pathway is usually involved in stress and inflammatory response (Coulthard et al., 2009) and has been shown to regulate NLRP3 inflammasome activation (Rajamäki et al., 2016). From the database of traditional Chinese medicine, we found that multiple target genes of mulberry active compounds are involved in the MAPK signal pathway. We speculated that mulberry extract may regulate the activation of NLRP3 inflammatory bodies of macrophages via p38 MAPK pathway. Indeed, our data supports this hypothesis by showing that the protein levels of NLRP3, phosphorylated p38 MAPK, and IL‐1β all decrease upon treatment of mulberry extract, making mulberry extract a potential treatment for not only restenosis but also other inflammation‐related diseases.

In summary, our study uncovers the elevated expression of NLRP3 in restenosis artery wall, making it a novel therapeutic target for the disease. More importantly, we found that mulberry extract plays multiple regulatory roles in the formation of restenosis. It not only inhibits the inflammation in foam cells by suppressing p38 MAPK signaling pathway mediated inflammasome activation but also stimulates the ABCA1/ABCG1 mediated cholesterol efflux of foam cells to decrease their formation. Therefore, mulberry extract is a promising all natural and highly effective treatment for neoatherosclerosis and restenosis.

## CONFLICT OF INTEREST STATEMENT

The authors declare that they have no known competing financial interests or personal relationships that could have appeared to influence the work reported in this paper.

## Data Availability

The data and materials could be acquired from the corresponding authors by reasonable request.
